# Vitamin A status regulates glucocorticoid availability in Wistar rats: consequences on cognitive functions and hippocampal neurogenesis?

**DOI:** 10.3389/fnbeh.2014.00020

**Published:** 2014-02-04

**Authors:** Damien Bonhomme, Amandine M. Minni, Serge Alfos, Pascale Roux, Emmanuel Richard, Paul Higueret, Marie-Pierre Moisan, Véronique Pallet, Katia Touyarot

**Affiliations:** ^1^INRA, Nutrition et Neurobiologie Intégrée (NutriNeuro), UMR 1286Bordeaux, France; ^2^University of Bordeaux, Nutrition et Neurobiologie Intégrée (NutriNeuro), UMR 1286Bordeaux, France; ^3^INSERM, Biothérapie des Maladies Génétiques et Cancer, U1035Bordeaux, France

**Keywords:** Vitamin A status, retinoic acid, glucocorticoid, spatial memory, anxiety-like behavior, hippocampal neurogenesis, 11beta-Hydrosteroid dehydrogenase type 1, corticosteroid binding globulin

## Abstract

A disruption of the vitamin A signaling pathway has been involved in age-related memory decline and hippocampal plasticity alterations. Using vitamin A deficiency (VAD), a nutritional model leading to a hyposignaling of the retinoid pathway, we have recently demonstrated that retinoic acid (RA), the active metabolite of vitamin A, is efficient to reverse VAD-induced spatial memory deficits and adult hippocampal neurogenesis alterations. Besides, excess of glucocorticoids (GCs) occurring with aging is known to strongly inhibit hippocampal plasticity and functions and few studies report on the counteracting effects of RA signaling pathway on GCs action. Here, we have addressed whether the modulation of brain GCs availability could be one of the biological mechanisms involved in the effects of vitamin A status on hippocampal plasticity and functions. Thus, we have studied the effects of a vitamin A-free diet for 14 weeks and a 4-week vitamin A supplementation on plasma and hippocampal corticosterone (CORT) levels in Wistar rats. We have also investigated corticosteroid binding globulin (CBG) binding capacity and 11beta-Hydrosteroid Dehydrogenase type 1 (11β-HSD1) activity, both important modulators of CORT availability at the peripheral and hippocampal levels respectively. Interestingly, we show that the vitamin A status regulates levels of free plasma CORT and hippocampal CORT levels, by acting through a regulation of CBG binding capacity and 11β-HSD1 activity. Moreover, our results suggest that increased CORT levels in VAD rats could have some deleterious consequences on spatial memory, anxiety-like behavior and adult hippocampal neurogenesis whereas these effects could be corrected by a vitamin A supplementation. Thus, the modulation of GCs availability by vitamin A status is an important biological mechanism that should be taken into account in order to prevent age-related cognitive decline and hippocampal plasticity alterations.

## Introduction

The vitamin A, through its main metabolite retinoic acid (RA), plays a key role in cognitive functions and more specifically in anxiety-like behavior and hippocampus-dependent memory during adulthood (Lane and Bailey, 2005; McCaffery et al., 2006; Cai et al., 2010; Olson and Mello, 2010). A disruption of RA signaling pathway has been involved in age-related memory decline (Etchamendy et al., 2001; Mingaud et al., 2008). Interestingly, in these studies life-long nutritional vitamin A supplementation or RA treatment corrected memory deficits in aged rodents. The involvement of retinoids in the control of hippocampal plasticity, known to underlie spatial memory processing (Eichenbaum et al., 1999; Eichenbaum, 2004), has been largely demonstrated in Vitamin A Deficiency (VAD) models, a nutritional approach leading to a hyposignaling of RA pathway. Thus, VAD disrupted hippocampal long-term potentiation (Misner et al., 2001; Jiang et al., 2012), hippocampal neurogenesis (Jacobs et al., 2006) and induced spatial and relational memory deficits (Cocco et al., 2002; Etchamendy et al., 2003). Furthermore, we have recently demonstrated that VAD-induced hippoccampal neurogenesis alterations and spatial memory deficits could be corrected by RA treatment (Bonnet et al., 2008).

Yet, it is still not clear how the vitamin A status modulates plasticity and memory processes. On the one hand, it is now commonly accepted that RA regulates gene expression including plasticity-related genes through binding to specific nuclear receptors: retinoic acid receptors (RARs) or retinoid X receptors (RXRs) (Marill et al., 2003). It has been shown that VAD could alter hippocampal plasticity and functions through a hypoexpression of some retinoid receptors, which has been associated with decreased plasticity-related target gene expression (Etchamendy et al., 2003; Husson et al., 2003, 2004). On the other hand, it has recently been proposed that the stimulation of retinoid signaling pathway antagonizes glucocorticoid-mediated actions (Paez-Pereda et al., 2001; Aubry and Odermatt, 2009; Brossaud et al., 2013). Thus, the deleterious effects of VAD may also be explained by a more indirect action of vitamin A status on the availability of glucocorticoids (GCs) in the hippocampus.

The hippocampus is a prime target for GCs as it contains the highest number of GCs receptors that can modulate memory processes (Oitzl and de Kloet, 1992). High levels of circulating GCs as a consequence of chronic stress or aging are known as risk factors in the development of psychopathologies (de Kloet et al., 2005). Thus, prolonged exposure to an excess of corticosterone (CORT) in rodents can lead to hippocampal atrophy with a significant disbranching and shortening of apical dendrites (Magarinos and McEwen, 1995; McEwen, 1999; Krugers et al., 2010) and these hippocampal alterations have been correlated with memory impairments (Sousa et al., 2000; Sandi, 2003; Sandi et al., 2004; Joels and Krugers, 2007). A reduction in adult hippocampal neurogenesis after chronic CORT exposure is also associated with learning impairments (Montaron et al., 2006; Klempin and Kempermann, 2007; Yau et al., 2012).

The magnitude of CORT action in the rodent hippocampus is thought to be determined (i) by the activity of hippocampal 11β-Hydroxysteroid Dehydrogenase type 1 (11β-HSD1), an enzyme that regenerates active CORT within cells, and (ii) by free CORT circulating in the blood, delivered to the brain (Seckl, 1997) and regulated by corticosteroid binding globulin (CBG) (Breuner and Orchinik, 2002). Both the hyperactivity of hippocampal 11β-HSD1 and elevated plasma CORT, are correlated with impairments in hippocampal-dependent memory tasks during aging (Yau et al., 2001; Holmes et al., 2010; Yau and Seckl, 2012). Interestingly, the inhibitory effects of RA and vitamin A supplementation have been shown on the expression and the activity of 11β-HSD1, in differentiated C2C12 myotubes (Aubry and Odermatt, 2009), in obese rat liver (Sakamuri et al., 2011) but also in vitamin A-deficient LOU/C rats (Arvy et al., 2013). Indeed, in this latter study, the VAD-induced up-regulation of the hippocampal expression of 11β-HSD1 has been associated to an increased Hypothalamic-Pituitary-Adrenal (HPA) axis activity in basal and stress conditions which has been normalized by a RA treatment (Arvy et al., 2013). Finally, a RA treatment inhibits the hypersecretion of CORT in an experimental model of the Cushing's syndrome (Paez-Pereda et al., 2001) suggesting that it could be used as a successful treatment to reverse endocrine and cognitive alterations found in stress-related disorders.

Thus, these data show some antagonistic effects between GCs and retinoid pathways. Here, we hypothesize that the stimulation of the retinoid pathway could be a successful strategy to counteract the deleterious effects of an excess of GCs on hippocampal plasticity and functions. The effects of VAD and supplementation on hippocampus-dependent memory and anxiety-like behavior have been assessed. To clarify the molecular mechanisms underlying VAD-induced behavioral alterations, we have evaluated how vitamin A status could modulate GCs availability both at the peripheral and the hippocampal levels. Thus, we have demonstrated for the first time that VAD induced an elevated free CORT in the plasma and the hippocampus, a downregulation of plasma CBG binding capacity and a hyperactivity of hippocampal 11β-HSD1. Moreover, such deleterious effects are associated with spatial memory deficits, elevated anxiety, and decreased hippocampal neurogenesis in VAD rats, which could be corrected by a vitamin A supplementation. Thus, acting on vitamin A status could be a good strategy to prevent excess GCs-induced cognitive decline occurring with aging.

## Materials and methods

### Animals

Weaned male Wistar rats (3 weeks old) were purchased from Janvier (Le Genest Saint-Isle, France). They were housed two per cage in a room with a constant airflow system, controlled temperature (21–23°C), and a 12 h light/dark cycle. Rats were given *ad libitum* access to food and water and weighed twice a week. As in (Bonnet et al., 2008), 1 week prior to the beginning of behavioral experiments, all animals were housed individually until sacrifice. All experiments were performed in accordance with the European Communities Council Directives (86/609/EEC) and the French national Committee (87/848) recommendations, and have been approved by the Animal Care and Use Committee of Bordeaux under the N°50120169-A.

### Diet

At their arrival, the weaned rats were randomly assigned to two experimental groups: one group (*n* = 40) received a vitamin A-free diet (Laboratorio Piccionni, Italy), whereas the second one (*n* = 40) was fed with a control diet containing 5 IU retinol/g (INRA, Jouy-en-Josas). Subsequently, the weaned rats (*n* = 80) have been fed with a control diet containing 5 IU retinol/g or a vitamin A deficient diet (0 IU retinol/g) for 10 weeks. They are referred to as control rats (*n* = 40) and VAD rats (*n* = 40), respectively. Then, half of the vitamin A-deficient rats (*n* = 20) and half of the control rats (*n* = 20) have been supplemented with a vitamin A-enriched diet (20 IU retinol/g) for 4 weeks: they were referred to as VAD + Vit A and Control + Vit A, respectively while the other halves have been kept on their respective diets. The supplemented vitamin A diet (20 IU retinol/g) has been used, as it has been shown to be effective in reversing the VAD-related memory decline (Cocco et al., 2002).

### Experimental design

**Figure 1:**
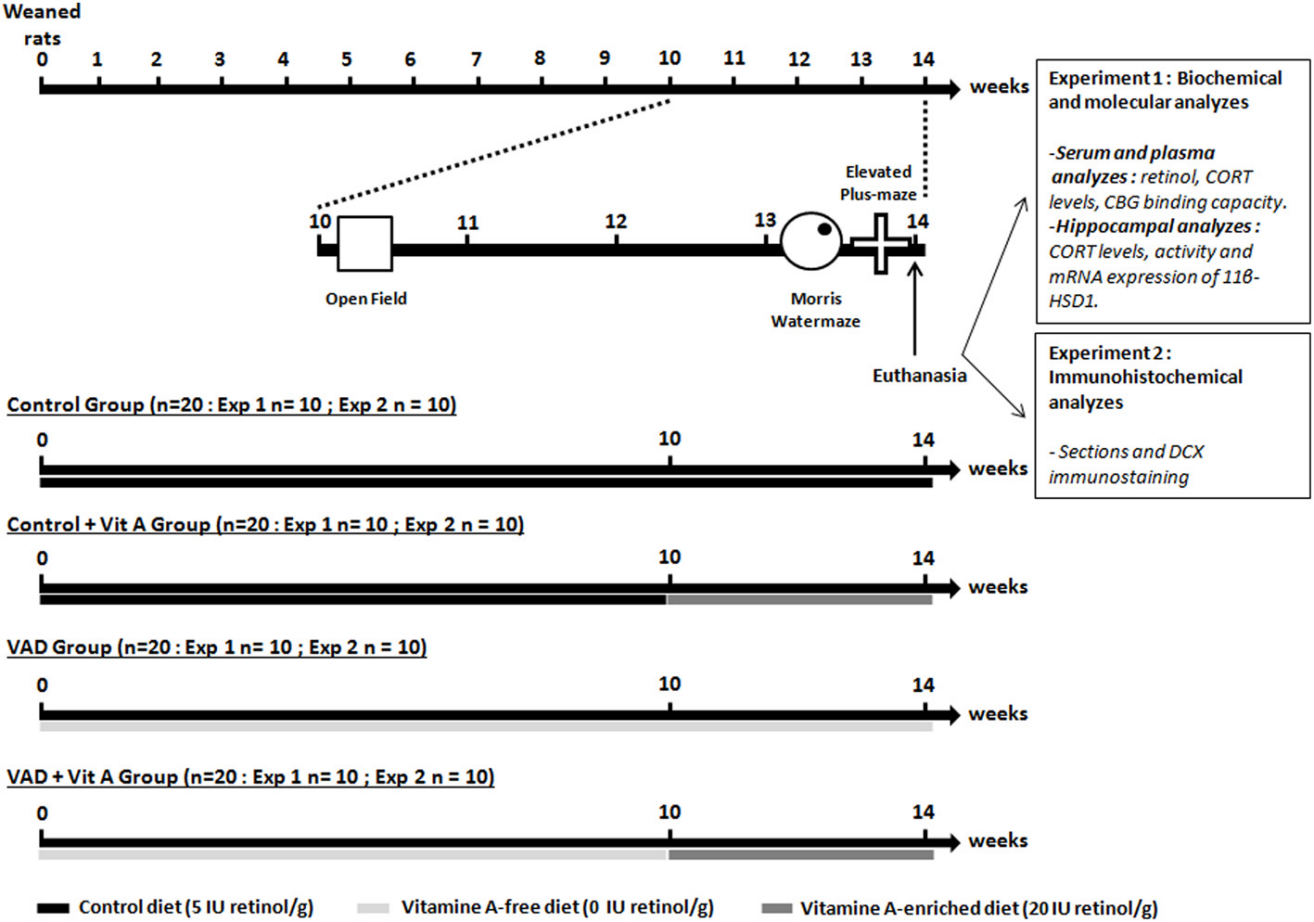
**Experimental design**. The weaned rats were fed with a control diet containing 5 IU retinol/g or a vitamin A-deficient diet for 10 weeks until the open-field test. From the 10thweek of VAD, half of the animals was supplemented with a vitamin A-enriched diet (20 IU retinol/g) for 4 weeks (Control, VAD, Control + Vit A, VAD + Vit A). After euthanasia, the first experiment (Exp 1) investigated the effects of 14 weeks of VAD and a 4-week vitamin A supplementation on retinoid and GCs status both at the peripheral levels (serum retinol, plasma CORT, CBG binding capacity) and the hippocampal levels (activity and expression of 11β-HSD1, CORT levels). These parameters were evaluated after the completion of spatial memory and anxiety-like behavior tasks. In the second experiment (Exp 2), we studied the effects of 14 weeks of VAD and a 4-week of vitamin A supplementation on hippocampal neurogenesis (immunostaining of DCX) evaluated after the behavioral tests. The arrows and the bars indicate the time scale and the diet received by the rats, respectively.

We have studied the effects of vitamin A status (deficiency and supplementation) on CORT availability at the plasma and the hippocampal levels and its impact on hippocampal plasticity and functions (Figure 1). After 10 weeks of diet, the two experimental groups (Controls *n* = 40, VAD *n* = 40) were tested in the open field test with a systematic characterization of locomotor reactivity to novelty. We could thus subdivide the two experimental groups and equilibrate their activity scores: half of the controls and half of the VAD rats (Controls, VAD, Control + Vit A, VAD + Vit A) have been supplemented during the next 4 weeks. Thirteen weeks after their arrival, rats were trained and tested in a Morris water maze spatial reference memory task followed by an elevated plus maze to assess their anxiety-like behavior. One day after the anxiety test, all groups were sacrificed in the morning; blood samples and hippocampi were collected for further biochemical and biomolecular analyzes (experiment 1) and immunohistochemical analyzes (experiment 2).

### Behavioral tests

#### Open-field

This test measures the spontaneous locomotor activity of the animals on placement in a novel environment. The floor (1 × 1 m) is a white square in Plexiglas with walls (45 cm) made in PVC. The open field arena is thoroughly cleaned before each rat is tested. Ten weeks after their arrival, the locomotor activity of the rats was measured using the video tracking system containing an infrared video camera. Each animal was placed in the center of the apparatus and the total distance travelled by the animals was recorded during 10 min (Videotrack, Viewpoint, Lyon, France). A low light intensity (60 lux) was used to limit high anxiety levels and to obtain normal amount of exploration levels.

#### Spatial reference memory in the morris water maze

Rats were tested in a Morris water maze (180 cm diameter, 60 cm high) filled with water (22 ± 1°C) with a spatial reference memory protocol as previously described in Bonnet et al. (2008). Briefly, 13 weeks after their arrival, animals were required to locate a submerged platform by using distal extramaze cues. They were trained four trials a day (90 s with an intertrial interval of 60 s, starting from three different start points randomized every day) for 7 consecutive days. As the latency to reach the hidden platform is dependent on the swimming speed, the distance covered to reach the platform is a more appropriate measure and has been chosen as a good index of the acquisition rate for spatial learning in the water maze. On day 8 (at 14 weeks of VAD), spatial memory was evaluated by the percentage of time spent in the quadrant where the platform was located during training (target quadrant). After the probe trial, on day 9, animals were trained for 4 trials to find a visible platform. Two VAD rats were excluded from the experiment due to failure to search for the platform during the acquisition phase (floating).

#### Elevated plus-maze

Two days after the end of the water maze, rats were tested for anxiety-like behavior in the elevated-plus maze. The elevated plus-maze apparatus consisted of two opposite open arms (50 × 10 cm) and two opposite enclosed arms (50 × 10 cm) emanating from a common central platform (10 × 10 cm) to form a plus shape. The apparatus was elevated 50 cm above the floor. The luminosity conditions were the same than that of the open-field test (60 lux). Each rat was placed on the central platform with his head facing an open arm. The anxiety-related behaviors (total time and percentage of time spent in open arms as indexes of anxiety (Walf and Frye, 2007) of all animals and their global locomotor activity were recorded for a period of 10 min by a video camera (Videotrack, Viewpoint, Lyon, France).

### Tissue preparation

One day after the elevated plus maze, rats were transferred to a room adjacent to the laboratory, were euthanized with isoflurane, and decapitated within 3 min to avoid the effects of euthanasia on plasma CORT levels.

As described in Figure 1 (experiment 1), trunk blood was collected immediately in order to measure serum retinol, plasma CORT, and CBG binding capacity. Trunk blood was then centrifuged to obtain serum samples (1500 g for 15 min) and plasma samples (1500 g for 10 min in tubes containing 10% EDTA). The supernatant was collected and stored until assay at −20°C and at −80°C respectively.

Ten brains per group were randomly assigned for later measurements: i.e., 40 for biochemical and PCR analyzes (Figure 1, experiment 1) and 40 for DCX immunohistochemistry (Figure 1, experiment 2). In order to measure hippocampal activity of 11β-HSD1 and its mRNA expression by quantitative RT-PCR and to evaluate hippocampal levels of CORT, the hippocampi from both cerebral hemispheres were rapidly removed, frozen in liquid nitrogen, and then stored at −80°C until assay. In order to analyze hippocampal neurogenesis by immunohistochemistry, the dissected brains were washed with 0.9% sodium chloride and emerged in 4% paraformaldehyde. After a 3-week postfixation period, 50 μm coronal sections were cut on a vibratome (Leica).

### Serum retinol

Serum retinol was assayed by HPLC according to a previously described method (Biesalski et al., 1983).

### Plasma CORT and CBG analyzes

Total and free CORT levels and CBG binding capacity were measured in the same plasma samples.

Total plasma CORT concentration was measured by RIA (see Richard et al., 2010 for details). Briefly, after steroid extraction of plasma samples with absolute ethanol, total CORT was measured by competition between cold CORT and ^3^H-CORT by a specific anti-CORT antibody provided by Dr H.Vaudry (University of Rouen, France). The sensitivity of this assay is around 5 ng/mL.

Free plasma CORT concentration was measured by isotopic dilution and plasma ultrafiltration using Centrifree filter device (YM membranes 30 K, Millipore, France) as in (Richard et al., 2010), using 100 μL of plasma. Free CORT fraction was calculated as the ratio of counts per minute (cpm) filtrate (free CORT)/cpm total CORT.

CBG maximum binding capacity (Bmax) and Kd were measured with a saturation curve and Scatchard analysis as described in Richard et al. (2010) using a standard curve of tritiated CORT up to 64 nM.

### CORT and 11β-HSD1 analyzes in the hippocampus

Half of the microdissected hippocampi was homogenized on ice in 1 mL of buffer (1.37 M Glycerol, 300 mM NaCl, 1 mM EDTA, 50 mM Tris, 1X Phosphatase Inhibitor Cocktail, 2 mM NaOV, 1 mM NaF; pH = 7.7). The total protein content of the homogenate was determined with a BC Assay kit (Uptima, Montluçon, France).

Hippocampal levels of CORT were measured by an enzyme immunoassay commercial kit (Correlate-EIA; Assay Designs, Ann Arbor, MI) from homogenates containing a final protein concentration of 6 mg/mL. This assay was chosen for his high sensitivity, allowing the detection of low levels of CORT (around 18.6 pg/mL).

Hippocampal activity of 11β-HSD1 was also measured from the same homogenates. *In vivo*, 11β-HSD1 catalyzed the conversion of inactive 11-dehydroCORT to CORT. According to (Moisan et al., 1990), dehydrogenase activity was measured by quantifying the conversion of CORT (B) to 11-dehydroCORT (A). 0.5 mg/mL of total protein were incubated at 37°C for 1 h with 12 nM ^3^H-CORT as substrate (specific activity: 78.1 Ci/mmol, PerkinElmer) and an excess (400 μM) of the enzyme-specific cofactor NADP. After incubation, steroids were extracted by addition of ethyl acetate, separated by thin-layer chromatography on silica gel plates (TLC Silica Gel 60 F254, VWR) using a mixture of chloroform and ethanol (92:8). Then, ^3^H-CORT and ^3^H-dehydroCORT were quantified with a β-Imager apparatus and 11β-HSD1 activity was expressed as the percentage conversion of ^3^H-CORT (B) to ^3^H-dehydroCORT (A).

### Real-time PCR analyzes of retinoid target gene expression in the hippocampus

The other half of the hippocampi was used to measure gene expression. RNA extraction was conducted using TRIzol reagent (Invitrogen, Saint Aubin, France) according to the manufacturer's instructions. The integrity of the purified RNA was verified using the RNA 6000 Nano LabChip kit in combination with the 2100 Bioanalyzer (Agilent Technologies). The concentrations of RNA were determined by using a Nanodrop ND-1000 (Labtech). Using oligodT and random primers (Promega, Charbonnières les bains, France), cDNA was synthesized from 1 μg of RNA with ImPromII reverse transcriptase (Promega, Charbonnières les Bains, France) according to the manufacturer protocol. The real-time PCR was performed using the LightCycler 480 system with a 96-well format (Roche Diagnostics) in a volume of 20 μL, containing 1X LightCycler 480 SYBR Green I Master solution, 0.5 μM of each primer and 6 μL of cDNA. The forward and reverse primer sequences for the 11β-HSD1 and β-microglobulin (BMG) that has been used as a house-keeping gene, was the following: 11β-HSD1-f: AAAATACCTCCTCCCCGTCCTG; 11β-HSD1-r: TCTCTTCCGATCCCTTTGCTG; BMG-f: GCCCAACTTCCTCAACTGCTACG; BMG-r: GCATATACATCGGTCTCGGTGGG. The results were expressed as the target/reference ratio divided by the target/reference ratio of the calibrator.

### Immunohistochemistry

Free-floating sections were processed with a standard immunohistochemical procedure (Lemaire et al., 2006). A one–in-ten section was treated for doublecortin (DCX) immunoreactivity using a goat polyclonal antibody (1:1000, Santa Cruz Biotechnology) and a biotinylated donkey anti-goat secondary antibody (1:200, Amersham). All sections were processed in parallel, and immunoreactivities were visualized by the biotin-streptavidin technique (ABC kit, Dako) by using 3,3-diaminobenzidine as chromogen. The number of immunoreactive (IR) cells in the left Dentate Gyrus (DG) was estimated by using a modified version of the optical fractionators method with a systematic random sampling of every 10 sections along the rostrocaudal axis of the DG. On each section, IR cells in the granular and subgranular layers of the DG were counted with a 100 × microscope objective (Lemaire et al., 2006). All results were expressed as the total number of DCX-IR cells in the whole DG.

### Statistical analysis

Locomotor activity was analyzed by a One-Way ANOVA (effect of deficiency). Reference memory, elevated-plus maze test, DCX immunohistochemistry, biochemical, and PCR data were analyzed using a Two-Way ANOVA (effect of deficiency and supplementation) followed by a *post-hoc* Fisher PLSD test. Body weight gain, spatial learning, and swim speed data were analyzed using a Three-Way ANOVA with repeated measures (effect of deficiency, supplementation and days or weeks) followed by a *post-hoc* Fisher PLSD test. All results were expressed as mean ± SEM.

## Results

### Effects of VAD on locomotor activity in the open-field test

The same diet protocol was used as in a previous study that has shown that the consumption of the vitamin A-free diet for 10 weeks induces a time course vitamin A depletion of the liver store in rats, leading to a decreased serum retinol concentration (Husson et al., 2003). The impact of vitamin A deficiency on locomotor activity in response to novelty was evaluated in the open-field test. The ANOVA on total distance revealed no significant difference between the control and VAD groups [7253.47 ± 523.59 cm vs. 7755.9 ± 546.09 cm; *F*_(1, 35)_ = 2.42, n.s.] indicating that a 10-week VAD does not induce alterations in global locomotor activity. The levels of activity scores were used to equilibrate the groups receiving or not a vitamin A supplementation (at 10 weeks, Control: 7481.9 ± 319.2 cm; Control + Vit A: 7025.1 ± 321.8 cm; VAD: 7760.9 ± 304.8 cm; VAD + Vit A: 7751 ± 290.2 cm).

### Effects of VAD and vitamin A supplementation on body weight and serum retinol concentration

In order to control the vitamin A status of the animals, we measured the body weight gain over 14 weeks and the serum retinol concentration after 14 weeks of diet.

**Figure 2:**
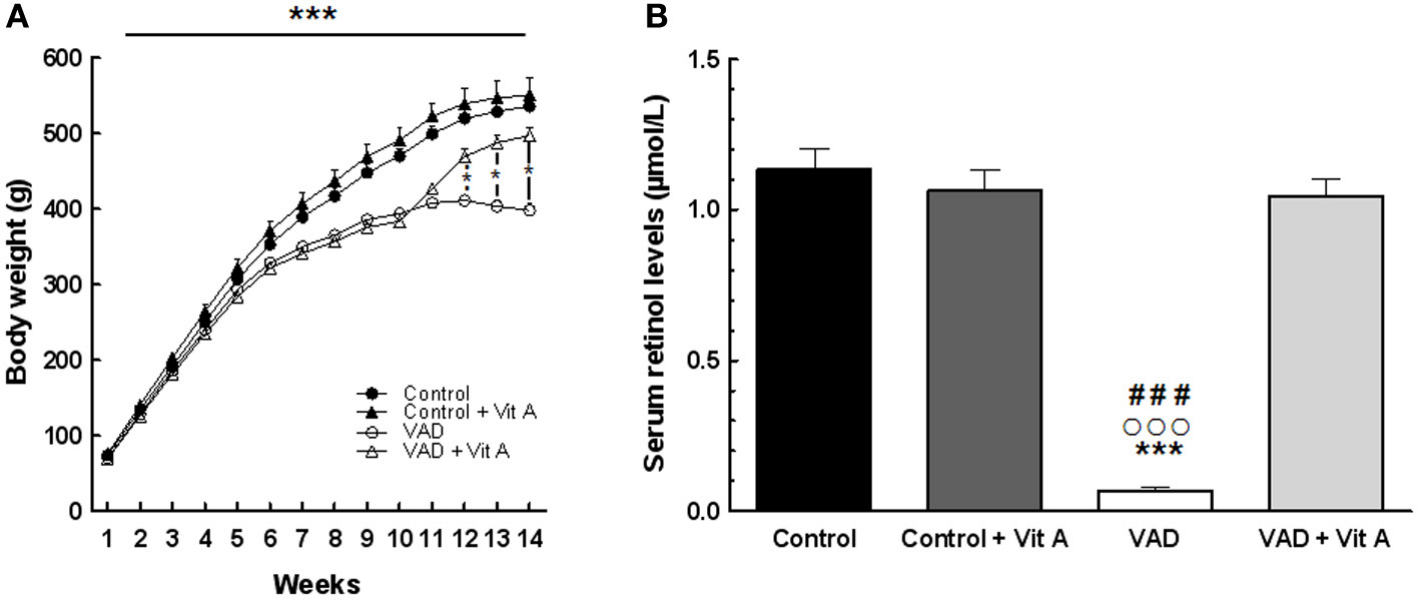
**Effects of 14 weeks of VAD and vitamin A supplementation on body weight gain (A) and serum retinol concentration (B). (A)** The growth of the vitamin A deprived rats slows down after 10 weeks and reaches a plateau until the 14th week of VAD. This effect is counteracted by vitamin A supplementation. **(B)** VAD decreases serum retinol levels and 4 weeks of supplementation normalize this level. ^*^p < 0.05 vs. VAD + Vit A; ^***^p < 0.001 vs. Control; ^###^*p* < 0.001 vs. Control + Vit A; °°°*p* < 0.001 vs. VAD + Vit A by Three-Way and Two-Way ANOVA, respectively followed by Fischer's *post-hoc* tests. *n* = 8–10 per group.

A Two-Way ANOVA on body weight over the 10 weeks of VAD revealed a highly significant effect of deficiency [*F*_(1, 35)_ = 32.69, *p* < 0.001] and a strong interaction deficiency × weeks [*F*_(9,315)_ = 52, *p* < 0.001]. Indeed, the Figure 2A indicates that the growth of the vitamin A-deprived rats reached a plateau after 10 weeks of VAD and then stabilized their body weight until the 14th week whereas control rats kept on gaining weight. However, a vitamin A supplementation during 4 weeks was sufficient to induce a significant increase of body weight in VAD rats while it did not affect the weight of control rats [Three-Way ANOVA between 11 and 14 weeks of VAD, deficiency × supplementation × weeks: *F*_(3, 99)_ = 52.20, *p* < 0.001]. On the 14th week, VAD rats that had been supplemented for 4 weeks had a similar weight as control rats (Fisher's *post-hoc*, *p* > 0.05, between VAD + Vit A and Controls).

The analysis of serum retinol levels was performed at 14 weeks of VAD in order to control for the vitamin A status for each group (Figure 2B). The ANOVA on serum retinol levels revealed a significant effect of deficiency [*F*_(1, 33)_ = 88.68, *p* < 0.001], a significant effect of supplementation [*F*_(1, 33)_ = 62.47, *p* < 0.001] with an interaction deficiency × supplementation [*F*_(1, 33)_ = 82.74, *p* < 0.001]. A significant reduction (−90%) in serum retinol concentration was observed in VAD rats (0.069 ± 0.001 μmol/L) relative to control rats (1.133 ± 0.068 μmol/L) (Fisher's *post-hoc*, *p* < 0.001). Four weeks of a vitamin A-enriched diet restored the serum retinol level in VAD rats (1.046 ± 0.054 μmol/L) (Fisher's *post-hoc*, *p* < 0.001, between VAD and VAD + Vit A) while no effect of supplementation was observed in control rats (Control + Vit A: 1.078 ± 0.061 μmol/L) (Fisher's *post-hoc*, *p* > 0.05, between Control and Control + Vit A).

### Effects of VAD and vitamin A supplementation on spatial learning and memory

The reference memory of rats was performed in the Morris water maze task.

**Figure 3:**
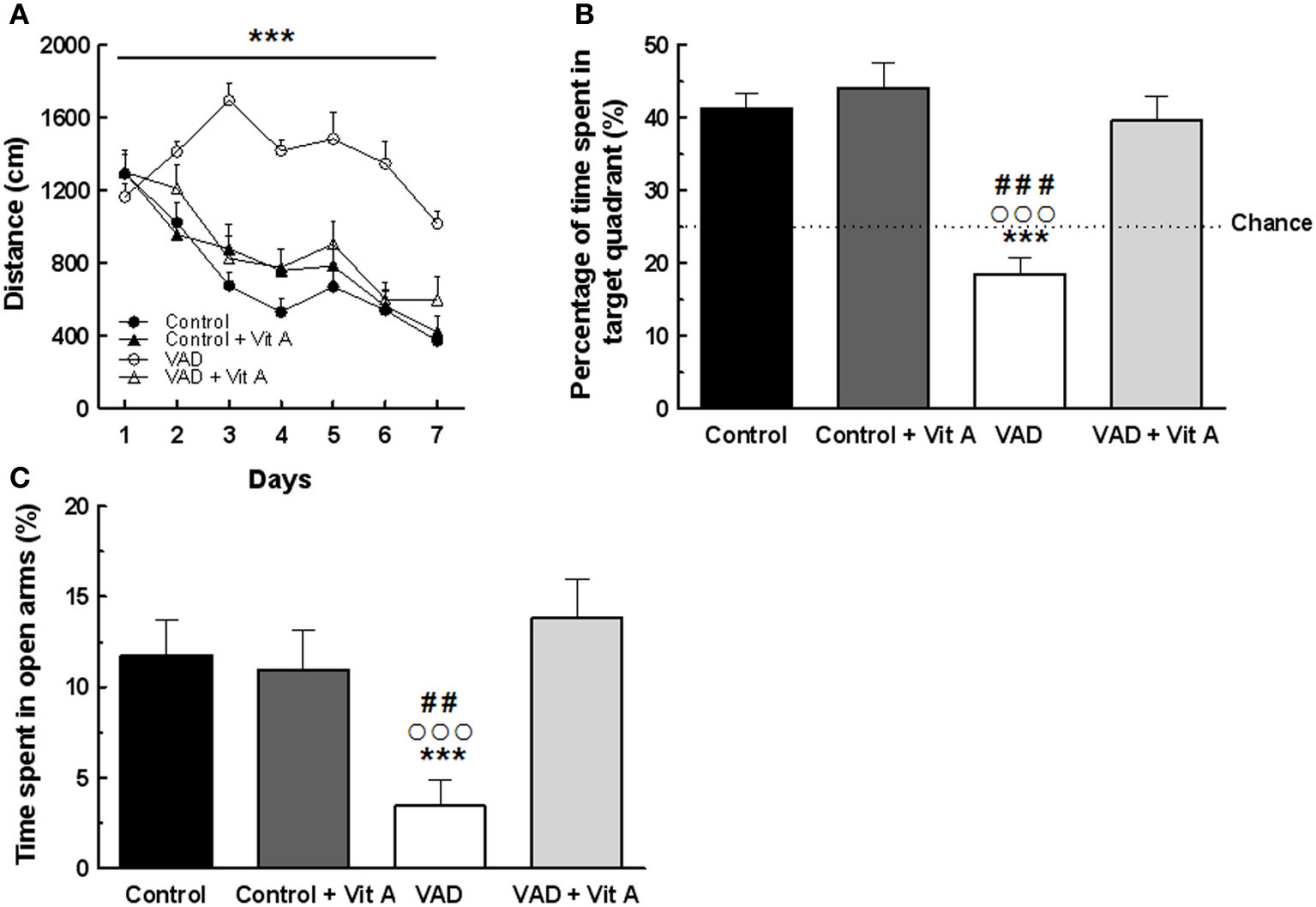
**Effects of 14 weeks of VAD and vitamin A supplementation on (A, B) spatial learning and memory and (C) anxiety-like behavior. (A)** Distance covered to reach the hidden platform over the 7 consecutive days of spatial learning (spatial learning; blocks of trials for each training day are averaged). **(B)** Percentage of time spent by rats in the target quadrant (Probe test). The dotted line corresponds to chance level (25%). VAD rats exhibit longer distance compared to controls to reach the platform during acquisition and they spent a percentage of time in the target quadrant around the chance level. The spatial learning and memory deficits are corrected by a 4-week vitamin A supplementation. **(C)** VAD decreases the percentage of time spent in open arms in plus-maze test suggesting an increased anxiety level in VAD rats which is normalized by supplementation. ^***^p < 0.001 vs. Control; ^##^*p* < 0.01 vs. Control + Vit A; ^###^*p* < 0.001 vs. Control + Vit A; °°°*p* < 0.001 vs. VAD + Vit A by Three-Way ANOVA (spatial learning) and Two-Way ANOVA followed by Fischer's *post-hoc* tests. *n* = 8–10 per group.

A Three-Way ANOVA of the distance to reach the platform over the seven training days (Figure 3A) revealed a significant effect of deficiency. Indeed, VAD rats travelled significantly longer distance to find the hidden platform than control rats, evidencing spatial learning impairments [*F*_(1, 32)_ = 29.03, *p* < 0.001]. Interestingly, the ANOVA revealed a significant effect of supplementation [*F*_(1, 32)_ = 8.97, *p* < 0.001] with an interaction days × deficiency × supplementation [*F*_(6, 192)_ = 3.96, *p* < 0.001] showing that a vitamin A-enriched diet could correct learning impairments of the VAD rats. However, the vitamin A supplementation had no effect on the spatial performances of the control rats.

Twenty four hours later, their spatial memory for the platform location was evaluated using a probe test (Figure 3B). A Two-Way ANOVA was performed on the percentage of time spent in the target quadrant and revealed a significant effect of deficiency [*F*_(1, 33)_ = 22.78, *p* < 0.001], and of supplementation [*F*_(1, 33)_ = 17.65, *p* < 0.001] with an interaction deficiency × supplementation [*F*_(1, 33)_ = 10.37, *p* < 0.001]. Unlike control rats, VAD rats did not look for the platform in the target quadrant as indicated by a percentage of time around the chance level (25%) suggesting that VAD induces spatial memory impairments (Fisher's *post-hoc* VAD vs. Control *p* < 0.001). Interestingly, a vitamin A supplementation normalized spatial memory performances in VAD rats, looking for the platform mainly in the correct quadrant, with a percentage of time similar to that observed in control rats (Fisher's *post-hoc* VAD + Vit A vs. VAD, *p* < 0.001; VAD + Vit A vs. Control, n.s.). However, the supplementation did not improve the performances of the control group (Fisher's *post-hoc* Control + Vit A vs. Control, n.s.).

Rats from all groups performed similarly in the control version of the water maze task with a visible platform indicating that learning differences were not due to differences in motor or visual capabilities, thigmotaxic behavior, or more generally to differences in health status (data not shown).

### Effects of VAD and vitamin A supplementation on anxiety-like behavior

The influence of 14 weeks of VAD and vitamin A supplementation on anxiety-like behavior was evaluated in the elevated plus-maze (Figure 3C). A Two-Way ANOVA on the percentage of time spent in the open arms revealed an effect of supplementation [*F*_(1, 33)_ = 5.73, *p* < 0.05] with an interaction deficiency × supplementation [*F*_(1, 33)_ = 7.78, *p* < 0.01]. Indeed, VAD animals spent less time in open arms (−70%) than control animals, (Fisher's *post-hoc* VAD vs. Control, *p* < 0.05) suggesting that they had higher anxiety levels but normal locomotor activity (no significant differences found on total travelled distance, data not shown). Interestingly, 4 weeks of vitamin A supplementation normalized the anxiety level of VAD rats (Fisher's *post-hoc* VAD vs. VAD + Vit A, *p* < 0.001) but had no effect on control animals (Fisher's *post-hoc* control vs. control + Vit A, n.s.).

### Effects of VAD and vitamin A supplementation on hippocampal neurogenesis

**Figure 4:**
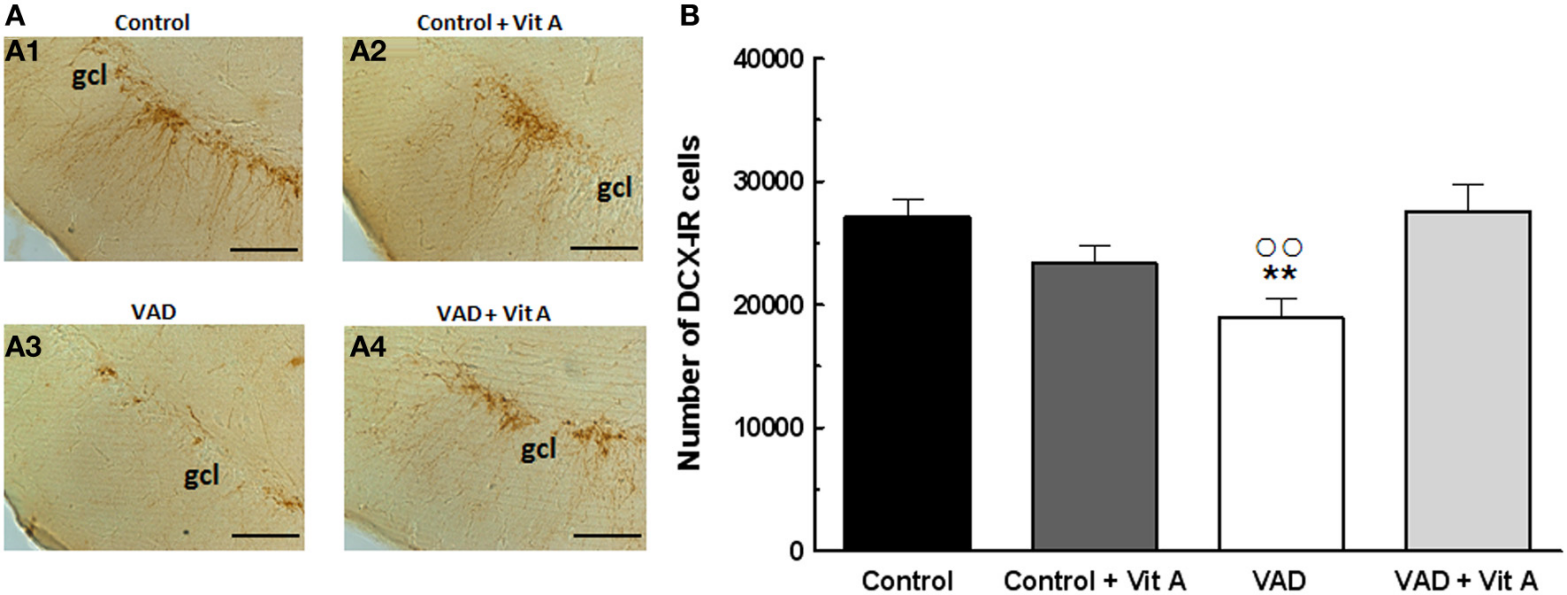
**Effects of 14 weeks of VAD and vitamin A supplementation on hippocampal neurogenesis. (A)** Images show the immunoperoxydase staining of DCX-IR cells in the DG granule cell layer in the different groups: Control (A1), Control + Vit A (A2), VAD (A3), VAD + Vit A (A4). **(B)** Number of DCX-IR cells in the DG granule cell layer. VAD induces a decrease in the number of IR cells which is corrected by vitamin A supplementation. ^**^p < 0.01 vs. Control; °°*p* < 0.01 vs. VAD + Vit A by Two-Way ANOVA followed by Fischer's *post-hoc* tests. Scale bar: 100 μm. gcl = granule cell layer. *n* = 10 per group.

The effects of retinoids on spatial memory have been proposed to be mediated, at least in part, by a modulation of hippocampal neurogenesis (Bonnet et al., 2008). As seen in Figure 4A, a quantitative analysis on the number of newly generated immature neurons revealed no effect of deficiency [*F*_(1, 35)_ = 0.99, n.s.] nor supplementation [*F*_(1, 35)_ = 2.4; n.s.] but an interaction deficiency × supplementation [*F*_(1, 35)_ = 6.14, *p* < 0.01] (Figure 4B). Thus, we showed that the number of DCX-IR cells was decreased in VAD rats (−25%) and this effect was compensated by a vitamin A supplementation that did not have any effect in control animals by itself (Fisher's *post-hoc* VAD vs. Control *p* < 0.01; VAD vs. VAD + Vit A *p* < 0.01, Control vs. Control + Vit A, n.s.).

### Effects of VAD and vitamin A supplementation on plasma glucocorticoid status

In order to verify whether vitamin A could act through the modulation of GCs, we investigated total and free plasma CORT and we also examined the plasma CBG binding capacity involved in the regulation of free CORT levels according to the vitamin A status.

**Figure 5:**
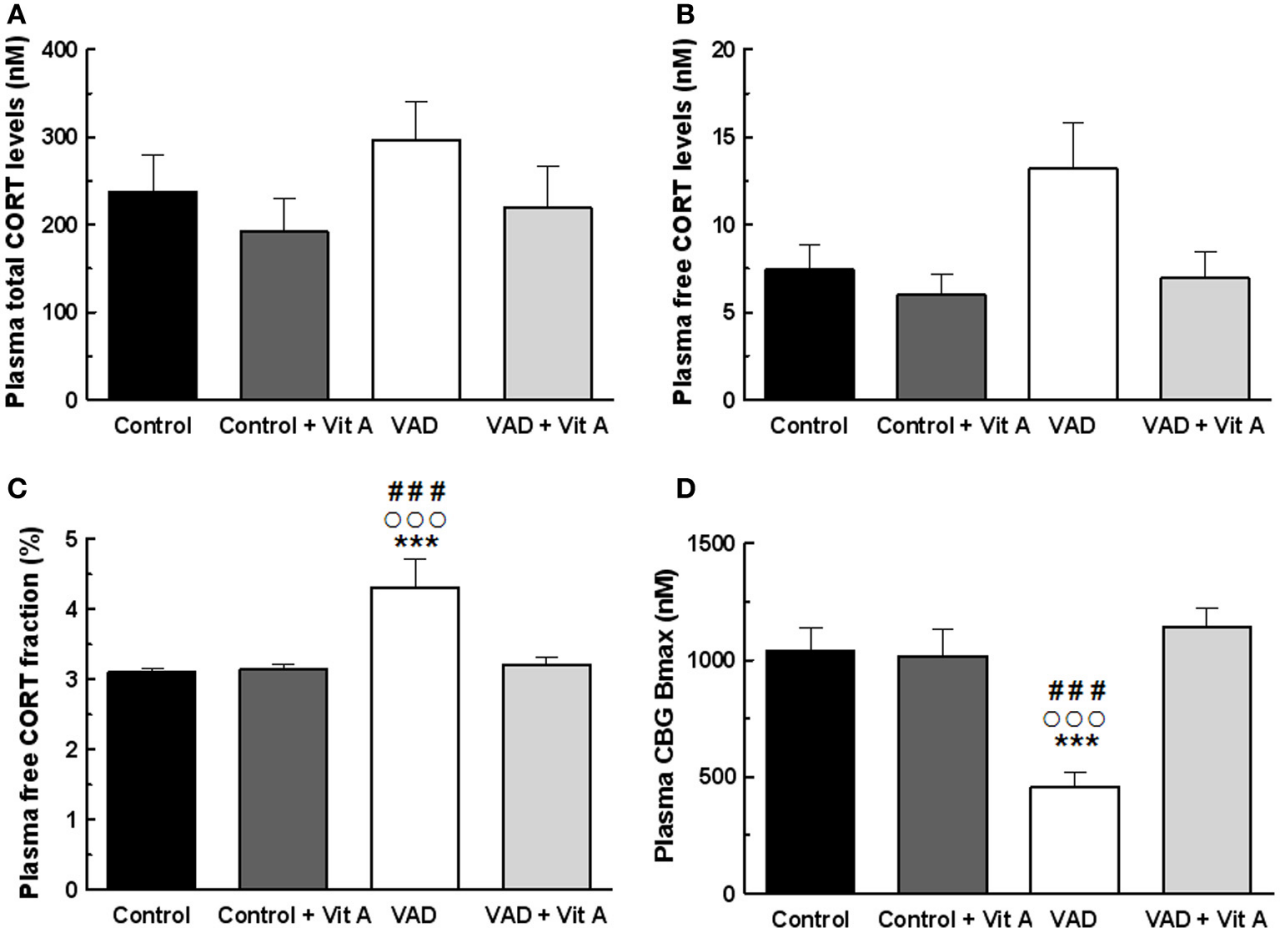
**Effects of 14 weeks of VAD and vitamin A supplementation on plasma glucocorticoid status. (A)** Total **(B)** free plasma CORT levels and **(C)** free plasma CORT fraction measured by RIA. While no significant effect on total CORT levels is detected between all groups, VAD increases free CORT fraction and vitamin A supplementation restores these levels. **(D)** CBG binding capacity measured by RIA. VAD rats exhibit lower plasma CBG Bmax compared to controls whereas vitamin A supplementation restores CBG binding capacity. ^***^p < 0.001 vs. Control; °°°*p* < 0.001 vs. VAD + Vit A; ^###^*p* < 0.001 vs. Control + Vit A by Two-Way ANOVA followed by Fischer's *post-hoc* tests. *n* = 9 per group.

No significant differences in the total plasma CORT concentration (Figure 5A) were found but the ANOVA on free plasma CORT levels (Figure 5B) revealed a deficiency tendency [*F*_(1, 32)_ = 3.59, *p* = 0.06] and a significant supplementation effect [*F*_(1, 32)_ = 4.72, *p* < 0.05] without deficiency × supplementation interaction [*F*_(1, 32)_ = 1.90, *p* = 0.17]. Interestingly, the ANOVA on plasma free CORT fraction (Figure 5C) indicated a strong deficiency effect [*F*_(1, 32)_ = 8.48, *p* < 0.01], a supplementation effect [*F*_(1, 32)_ = 5.79, *p* < 0.05] with a significant deficiency × supplementation interaction [*F*_(1, 32)_ = 6.54, *p* = 0.01]. Thus, free CORT fraction was increased in VAD rats (+38%) compared to controls (Fisher's *post-hoc* VAD vs. Control, *p* < 0.001; VAD: 4.3 ± 0.4% > Control: 3.1 ± 0.06%). The supplementation normalized the level of free CORT fraction in VAD rats (Fisher's *post-hoc* VAD vs. VAD + Vit A: 3.2 ± 0.09%, *p* < 0.001), but had no effect in control rats (Fisher's *post-hoc* Control vs. Control + Vit A: 3.13 ± 0.07%, n.s.).

As total circulating CORT is largely bound to CBG, we examined the importance of plasma CBG in regulating free plasma CORT under deficiency and supplementation conditions. The ANOVA on CBG Bmax concentrations (Figure 5D) showed significant effects of deficiency and of supplementation [*F*_(1, 32)_ = 6.15, *p* < 0.05; *F*_(1, 32)_ = 13.1, *p* = 0.001, respectively] with a strong deficiency × supplementation interaction [*F*_(1, 32)_ = 14.90, *p* < 0.001]. Fisher's *post-hoc* analyzes, revealed that the VAD diet significantly decreased CBG Bmax (−55%; VAD: 459.5 ± 62.4 nM vs Control: 1038 ± 99.3 nM, *p* < 0.001). Moreover, the level of CBG Bmax in VAD rats was normalized by the vitamin A supplementation (Fisher's *post-hoc* VAD vs. VAD + Vit A: 1142.61 ± 82.5 nM, *p* < 0.001) but was not modified in control rats. As observed for Bmax, the ANOVA on CBG Kd indicated significant effects (data not shown).

### Effects of VAD and vitamin A supplementation on hippocampal glucocorticoid status

We explored the possibility that 14 weeks of VAD and 4 weeks of vitamin A supplementation could modulate GCs availability in the hippocampus. Thus, activity and gene expression of 11β-HSD1 but also CORT levels were studied in the hippocampus.

**Figure 6:**
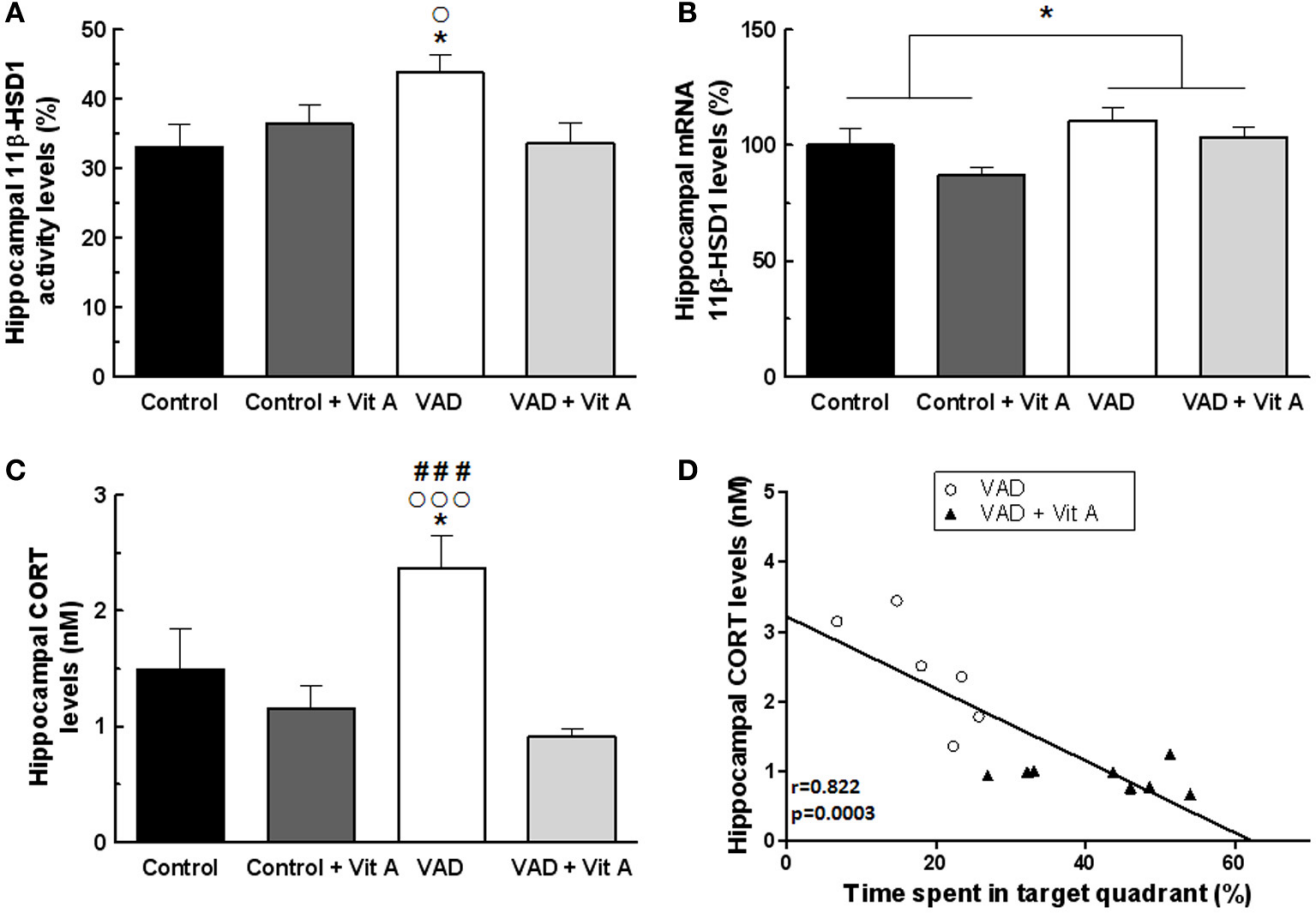
**Effects of 14 weeks of VAD and vitamin A supplementation on (A) activity and (B) mRNA expression of hippocampal 11**β-**HSD1, (C) hippocampal CORT concentration. (A)** The hippocampal 11β-HSD1 activity is increased in VAD rats and is restored by vitamin A supplementation. **(B)** Similar effects are observed on the normalized ratio of 11β-HSD1 mRNA expression. **(C)** VAD induces elevated levels of hippocampal CORT that are normalized by vitamin A supplementation. **(D)** Correlation analyzes. Hippocampal CORT levels negatively correlates with spatial memory in the probe test (*r* = 0.822, *p* = 0.0003). ^*^p < 0.05 vs. Control; °*p* < 0.05 vs. VAD + Vit A; °°°*p* < 0.001 vs. VAD + Vit A; ^###^*p* < 0.001 vs. Control + Vit A by Two-Way ANOVA followed by Fischer's *post-hoc* tests. *n* = 7–9 per group.

The activity of 11β-HSD1 within the hippocampus was not affected by the deficiency (Figure 6A). [*F*_(1, 29)_ = 1.81, n.s.] nor by the supplementation [*F*_(1, 29)_ = 1.39, n.s.] but there was a significant deficiency × supplementation interaction [*F*_(1, 29)_ = 5.28, *p* < 0.05]. Indeed, the activity of 11β-HSD1 was significantly increased in VAD rats (Fisher's *post-hoc* VAD vs. Control *p* < 0.05; VAD: 43.7 ± 2.5% > control: 33 ± 3.2%) and was normalized by vitamin A supplementation (Fisher's *post-hoc* VAD vs. VAD + Vit A *p* < 0.05; VAD: 43.7 ± 2.5% > VAD + Vit A: 33.5 ± 2.9%). However, this supplementation did not have any effect in control rats (Fisher's *post-hoc* Control vs. Control + vit A, n.s.). Thus, we checked whether this increased 11β-HSD1 activity in VAD rats could be due to an increase in mRNA expression. The ANOVA on hippocampal mRNA expression of 11β-HSD1 (Figure 6B) showed a deficiency effect indicating an increased level of 11β-HSD1 mRNA expression in rats submitted to VAD compared to control rats [*F*_(1, 29)_ = 6.52, *p* = 0.01]. Moreover, the vitamin A supplementation tended to decrease the levels of 11β-HSD1 expression in the hippocampus [*F*_(1, 29)_ = 3.59, *p* = 0.06].

As 11β-HSD1 catalyses the regeneration of active GCs within cells, we measured CORT levels in the hippocampus (Figure 6C). Whereas no effect of deficiency was observed, [*F*_(1, 29)_ = 1.61, n.s.], the ANOVA revealed a significant supplementation effect [*F*_(1, 29)_ = 12.85, *p* = 0.001] and a deficiency × supplementation interaction [*F*_(1, 29)_ = 5.04, *p* < 0.05] suggesting that VAD rats have a significant elevation of hippocampal CORT levels (+53.9%) compared to controls (Fisher's *post-hoc* VAD vs. Control *p* < 0.001; VAD: 2.37 ± 0.28 nM > Control: 1.49 ± 0.34 nM). Interestingly, the vitamin A supplementation normalized these CORT levels in VAD rats (Fisher's *post-h*oc VAD vs. VAD + Vit A *p* < 0.001; VAD: 2.37 ± 0.28 nM > VAD + Vit A: 0.91 ± 0.06 nM) but had no effect in control rats (Fisher's *post-h*oc Control vs. Control + Vit A *p* < 0.001; Control: 1.49 ± 0.34 nM ≈ Control + Vit A: 1.16 ± 0.19 nM).

As shown in Figure 6D, there was a strong negative correlation between hippocampal CORT levels and spatial memory (*r* = 0.82, *p* < 0.001) suggesting that the higher the level of hippocampal CORT, the poorer the spatial memory of the rats.

## Discussion

Our data demonstrate for the first time that vitamin A status can regulate GCs availability at both peripheral and hippocampal levels. Thus, the excess of free plasma and hippocampal CORT in VAD rats, which is associated with a decreased binding capacity of plasma CBG and a hyperactivity of hippocampal 11β-HSD1, could contribute to the appearance of spatial memory deficits, elevated anxiety-like behavior and hippocampal neurogenesis alterations observed after 14 weeks of VAD. Interestingly, such effects could be limited by a 4-week vitamin A supplementation.

### Vitamin A status regulates plasma CORT levels through CBG binding capacity

Prolonged exposure to elevated GCs during life has been hypothesized to contribute to the decline of hippocampal plasticity and functions (Cameron and Gould, 1994; Lupien et al., 1998; Yau and Seckl, 2012). It has recently been found that CBG would play a critical role in the deliverance of GCs to the brain by impacting on memory retrieval (Breuner and Orchinik, 2002; Minni et al., 2012). Indeed, CBG acts as a buffer of most systemic GCs by limiting the amount of circulating free hormones that are active on their receptors (Moisan, 2010; Richard et al., 2010). In order to better understand the mechanisms by which the hippocampal availability of GCs could be modulated by vitamin A status, we have studied total plasma CORT levels, free plasma CORT levels and CBG binding capacity, measured under resting conditions (in the morning). Neither a vitamin A deficient diet nor a vitamin A supplemented diet affects total plasma CORT levels. On the contrary, free plasma CORT levels is modulated by vitamin A status with a significant effect on free plasma CORT fraction, higher in VAD rats compared to controls and normalized by a vitamin A supplementation. Moreover, our data suggest that the increased free plasma CORT levels in VAD rats can be directly related to the decreased CBG binding capacity. This link is reinforced by data on vitamin A supplementation that appeared efficient in normalizing both effects. Thus, these results evidence an antagonistic effect of vitamin A on plasma GCs status.

The effects of VAD on plasma GCs status has been recently reported by our team in LOU/C rats (Arvy et al., 2013) that are derived from the Wistar strain and described as a model of healthy ageing with decreased age-related memory deficits (Alliot et al., 2002). Indeed, an increase in HPA axis activity has been shown in VAD LOU/C rats leading to elevated total plasma CORT levels in basal and stress conditions, which were restored by RA treatment (Arvy et al., 2013). We have shown that VAD in Wistar rats can increase HPA axis activity leading to hypersecretion of basal free plasma CORT level that is normalized by a vitamin A supplementation. Moreover, total or free plasma CORT level of the control rats are not affected by the vitamin A supplementation. On the contrary, it has been shown that long term RA treatment induced a hyperactivation of HPA axis accompanied with increased basal plasma CORT levels in young Sprague Dawley rats (Cai et al., 2010). This discrepancy may be related to differences in the treatment used (RA injections vs. food supplementation) and the nutritional status (deficient rats vs. control rats). Indeed, chronic injections of high doses of RA are known to induce alterations in hippocampal plasticity and functions in young animals with a normal vitamin A status (Crandall et al., 2004). The nutritional intake of vitamin A in our young control rats does not modify serum retinol concentration, known to be tightly regulated. In our nutritional conditions, RA would be physiologically provided to the tissues. Thus, we can suggest that the counteracting effects of the supplemented diet on free plasma CORT levels would be efficient only in animals exhibiting a decreased vitamin A status with a retinoid hyposignaling.

### Vitamin A status regulates hippocampal CORT levels through 11β-HSD1 activity

Here, we demonstrate for the first time that vitamin A supplementation can counteract the elevation of hippocampal CORT levels in VAD rats, probably by normalizing the activity and expression of hippocampal 11β-HSD1. We have shown previously that vitamin A status can induce a modulation of GCs at systemic levels which probably impact on the hippocampal availability of GCs (Yau and Seckl, 2012). The magnitude of intracellular GCs action is also thought to be determined by the activity of 11β-HSDs (Seckl, 1997). Indeed, 11β-HSD1 regenerates active GCs from their inactive forms, in specific areas in the adult brain, such as the hippocampus, thereby effectively amplifying intracellular GC levels before they bind to MRs and/or GRs (Holmes et al., 2003). Our results show that VAD diet induces a hyperactivity of the hippocampal 11β-HSD1, probably resulting from gene overexpression since the amount of 11β-HSD1 mRNA increased in VAD rats. Moreover, the increased activity of this enzyme is associated with elevated hippocampal CORT concentration suggesting an amplification of GCs signaling pathway in VAD rats. Interestingly, these effects on hippocampal 11β-HSD1 and local levels of CORT in VAD rats are abolished by a vitamin A supplementation suggesting that this enriched diet could exert an inhibitory effect on GCs hippocampal signaling pathway. Consistent with these findings, it has been shown that RA treatment can reduce both the gene expression and the activity of 11β-HSD1 in C2C12 myotubes (Aubry and Odermatt, 2009). Moreover, the vitamin A supplementation can normalize elevated hepatic levels of 11β-HSD1 in obese rats (Sakamuri et al., 2011) and the overexpression of hippocampal 11β-HSD1 in VAD LOU/C rats (Arvy et al., 2013). These effects of vitamin A status on hippocampal 11β-HSD1 could directly be mediated through nuclear retinoid receptors, that have been shown to regulate negatively 11β-HSD1 expression *in vitro* models (Aubry and Odermatt, 2009). Thus, since VAD induces a decrease in brain mRNA expression and immunoreactivity of some retinoid receptors (Husson et al., 2004; Arfaoui et al., 2013), the overexpression of 11β-HSD1 in vitamin A deficient rats could result from this regulatory mechanism.

### Does vitamin A status impact on hippocampal plasticity and functions via glucocorticoids?

Vitamin A and its active metabolite RA, act on memory processes by modulating different aspects of hippocampal plasticity (Etchamendy et al., 2001, 2003; Cocco et al., 2002; Mingaud et al., 2008) including hippocampal neurogenesis (Jacobs et al., 2006; Bonnet et al., 2008; Goodman et al., 2012; Touyarot et al., 2013). We also show that a nutritional approach could be as effective as a pharmacological treatment (Bonnet et al., 2008) to correct spatial memory deficits and hippocampal neurogenesis alterations induced by VAD. Thus, in VAD enriched rats, the newly absorbed vitamin A from diet may be directly used by extra-hepatic tissue such as the brain (Ross et al., 2009) to increase local synthesis of RA which would allow to normalize retinoid signaling and to maintain normal hippocampal plasticity and functions. Interestingly, we have also evidenced a negative correlation between hippocampal CORT levels and spatial memory: the higher the hippocampal CORT levels, the poorer the spatial memory performance, suggesting that the excess of local GCs could result in memory deficits in VAD rats. Thus, this result sustains the hypothesis that GCs signaling pathway could mediate the deleterious effects of VAD on hippocampal plasticity and functions.

Consistent with our findings, higher plasma cortisol or CORT levels is associated with spatial memory impairments in humans and rodents, respectively (Issa et al., 1990; Yau et al., 1995; Lupien et al., 1998). Moreover, aged mice exhibiting an overactivation of 11β-HSD1 (Yau et al., 1995; Yau and Seckl, 2012) show similar memory impairments as our VAD rats. The involvement of 11β-HSD1 in age-induced memory deficits has been demonstrated in 11β-HSD1 KO mice (Yau et al., 2001, 2007) or by decreasing the activity of the enzyme using selective inhibitors (Sooy et al., 2010; Mohler et al., 2011). The improved spatial memory in aged 11β-HSD1 KO mice has been proposed to occur via reduced intracellular CORT levels altering the balance of corticoid receptor activation (Yau et al., 2011). Moreover, in aged animals and in VAD LOU/C rats hippocampal expression of GR is decreased, leading to alterations of hippocampus-induced negative feedback on HPA axis and an increased plasma CORT levels in these animals (Mizoguchi et al., 2009; Arvy et al., 2013). Since CORT concentration in target tissues as the hippocampus results from (i) free plasma CORT produced by HPA axis and (ii) local CORT synthesis by 11β-HSD1 (Yau and Seckl, 2012), both the normalization of hippocampal 11β-HSD1 activity and free plasma CORT fraction by vitamin A supplementation, could contribute to the correction of VAD-induced memory deficits. Finally, we show that VAD rats with spatial memory deficits are more anxious than control rats, and that vitamin A supplementation normalizes this anxiety trait. According to our results, the alteration of vitamin A status by chronic injections of high doses of RA has been shown to induce an increased plasma CORT level that has been associated with increased anxiety in the elevated plus maze (Cai et al., 2010). Thus, a normal vitamin A status would allow to maintain hippocampus-dependent memory integrity but also anxiety-related responses probably through a tight control of the CORT availability in the hippocampus.

Besides, excess of GCs are known to strongly inhibit adult hippocampal neurogenesis (Cameron and Gould, 1994; Gould and Tanapat, 1999). Vitamin A supplementation may thus limit their negative effects and maintain normal neurogenesis. Indeed, the suppression of GCs secretion from mid-life by adrenalectomy increases neurogenesis in old animals and prevents the emergence of age-related memory disorders (Montaron et al., 2006). Moreover, 11β-HSD1 is particularly expressed in the DG and increased hippocampal neurogenesis has been found in young 11β-HSD1 KO mice (Yau et al., 2007). Thus, the beneficial effects of a vitamin A supplementation on VAD-induced neurogenesis decline could be partly mediated by hippocampal GCs pathway. This hypothesis has been also comforted by the fact that RA minimizes the potentially deleterious effect of GCs on the decreased secretion of BDNF in hippocampal HT22 cells (Brossaud et al., 2013), a neurotrophin known to stimulate adult neurogenesis processes and to be involved in the reduction of neurogenesis during chronic exposure to GCs (Duman and Monteggia, 2006). Finally, glutamatergic mechanisms are thought to be also involved in stress-induced changes of hippocampal neurogenesis (Joels et al., 2004; Hunter et al., 2009). As it has been shown recently that VAD could alter hippocampal glutamatergic transmission (Zhang et al., 2011; Jiang et al., 2012), all of these endpoints provide potential targets for novel treatment strategies of chronic GCs-induced hippocampal plasticity alterations by nutritional factors.

Considerable progress has been made in controlling VAD worldwide (Bloem et al., 2002) and fortification programs have been shown repeatedly to be an effective food-based strategy to improve Vitamin A status (Souganidis et al., 2013). Our data show that a vitamin A supplementation could act on biochemical and molecular parameters but also cerebral plasticity and cognitive functions. However, we cannot exclude the possibility that a control diet with a lower vitamin A content would also be efficient to correct the observed deleterious effects of VAD. Indeed, future investigations and additional prevention efforts are still needed to better determine the dietary forms and/or the doses of vitamin A.

## Conclusion

Altogether, the present study demonstrates for the first time a modulation of hippocampal CORT availability by the vitamin A status. Indeed, the vitamin A supplementation normalizes the excess of CORT in VAD rats, (i) at the plasma level by regulating free CORT fraction and CBG binding capacity but also (ii) at the hippocampal level probably through a modulation of 11β-HSD1 activity. Moreover, the strong negative correlation found between the hippocampal CORT levels and the spatial memory performances suggests that the modulation of hippocampal CORT availability by the vitamin A status could be a neurobiological mechanism by which the retinoid pathway impacts on hippocampal neurogenesis and functions. Since same alterations of vitamin A metabolism and GCs availability occur during aging, this study supports the idea that a vitamin A supplementation could be a potent way to prevent age-related cognitive impairments by maintaining normal vitamin A and GCs status in seniors.

## Author contributions

Conceived and designed the experiments: Damien Bonhomme, Véronique Pallet, Paul Higueret and Katia Touyarot. Performed the experiments: Damien Bonhomme, Amandine M. Minni, Serge Alfos, Emmanuel Richard and Pascale Roux. Analyzed the data: Damien Bonhomme, Amandine M. Minni and Katia Touyarot. Wrote the paper: Damien Bonhomme, Amandine M. Minni, Marie-Pierre Moisan, Véronique Pallet and Katia Touyarot.

### Conflict of interest statement

The authors declare that the research was conducted in the absence of any commercial or financial relationships that could be construed as a potential conflict of interest.

## References

[B1] Alliot J. Boghossian S. Jourdan D. Veyrat-Durebex C. Pickering G. Meynial-Denis D. (2002). The LOU/c/jall rat as an animal model of healthy aging? J. Gerontol. A Biol. Sci. Med. Sci. 57, B312–B320 10.1093/gerona/57.8.B312 12145357

[B2] Arfaoui A. Lobo M. V. Boulbaroud S. Ouichou A. Mesfioui A. Arenas M. I. (2013). Expression of retinoic acid receptors and retinoid X receptors in normal and vitamin A deficient adult rat brain. Ann. Anat. 195, 111–121 10.1016/j.aanat.2012.06.006 23017197

[B3] Arvy N. Hamiani R. Richard E. Moisan M. P. Pallet V. (2013). Vitamin A regulates HPA axis status in LOU/C rats. J. Endocrinol. 219, 21–27 10.1530/JOE-13-0062 23847298

[B4] Aubry E. M. Odermatt A. (2009). Retinoic acid reduces glucocorticoid sensitivity in C2C12 myotubes by decreasing 11beta-hydroxysteroid dehydrogenase type 1 and glucocorticoid receptor activities. Endocrinology 150, 2700–2708 10.1210/en.2008-1618 19179438

[B5] Biesalski H. K. Ehrenthal W. Gross M. Hafner G. Harth O. (1983). Rapid determination of retinol (vitamin A) in serum by high pressure liquid chromatography (HPLC). Int. J. Vitam. Nutr. Res. 53, 130–137 6885272

[B6] Bloem M. W. Kiess L. Moench-Pfanner R. (2002). Process indicators for monitoring and evaluating vitamin A programs. J. Nutr. 132, 2934S–2939S 12221272 10.1093/jn/132.9.2934S

[B7] Bonnet E. Touyarot K. Alfos S. Pallet V. Higueret P. Abrous D. N. (2008). Retinoic acid restores adult hippocampal neurogenesis and reverses spatial memory deficit in vitamin A deprived rats. PLoS ONE 3:e3487 10.1371/journal.pone.0003487 18941534 PMC2567033

[B8] Breuner C. W. Orchinik M. (2002). Plasma binding proteins as mediators of corticosteroid action in vertebrates. J. Endocrinol. 175, 99–112 10.1677/joe.0.1750099 12379494

[B9] Brossaud J. Roumes H. Moisan M. P. Pallet V. Redonnet A. Corcuff J. B. (2013). Retinoids and glucocorticoids target common genes in hippocampal HT22 cells. J. Neurochem. 125, 518–531 10.1111/jnc.12192 23398290

[B10] Cai L. Yan X. B. Chen X. N. Meng Q. Y. Zhou J. N. (2010). Chronic all-trans retinoic acid administration induced hyperactivity of HPA axis and behavioral changes in young rats. Eur. Neuropsychopharmacol. 20, 839–847 10.1016/j.euroneuro.2010.06.019 20659790

[B11] Cameron H. A. Gould E. (1994). Adult neurogenesis is regulated by adrenal steroids in the dentate gyrus. Neuroscience 61, 203–209 10.1016/0306-4522(94)90224-0 7969902

[B12] Cocco S. Diaz G. Stancampiano R. Diana A. Carta M. Curreli R. (2002). Vitamin A deficiency produces spatial learning and memory impairment in rats. Neuroscience 115, 475–482 10.1016/S0306-4522(02)00423-2 12421614

[B13] Crandall J. Sakai Y. Zhang J. Koul O. Mineur Y. Crusio W. E. (2004). 13-cis-retinoic acid suppresses hippocampal cell division and hippocampal-dependent learning in mice. Proc. Natl. Acad. Sci. U.S.A. 101, 5111–5116 10.1073/pnas.0306336101 15051884 PMC387382

[B14] de Kloet E. R. Joels M. Holsboer F. (2005). Stress and the brain: from adaptation to disease. Nat. Rev. Neurosci. 6, 463–475 10.1038/nrn1683 15891777

[B15] Duman R. S. Monteggia L. M. (2006). A neurotrophic model for stress-related mood disorders. Biol. Psychiatry 59, 1116–1127 10.1016/j.biopsych.2006.02.013 16631126

[B16] Eichenbaum H. (2004). Hippocampus: cognitive processes and neural representations that underlie declarative memory. Neuron 44, 109–120 10.1016/j.neuron.2004.08.028 15450164

[B17] Eichenbaum H. Dudchenko P. Wood E. Shapiro M. Tanila H. (1999). The hippocampus, memory, and place cells: is it spatial memory or a memory space? Neuron 23, 209–226 10.1016/S0896-6273(00)80773-4 10399928

[B18] Etchamendy N. Enderlin V. Marighetto A. Pallet V. Higueret P. Jaffard R. (2003). Vitamin A deficiency and relational memory deficit in adult mice: relationships with changes in brain retinoid signalling. Behav. Brain Res. 145, 37–49 10.1016/S0166-4328(03)00099-8 14529804

[B19] Etchamendy N. Enderlin V. Marighetto A. Vouimba R. M. Pallet V. Jaffard R. (2001). Alleviation of a selective age-related relational memory deficit in mice by pharmacologically induced normalization of brain retinoid signaling. J. Neurosci. 21, 6423–6429 11487666 10.1523/JNEUROSCI.21-16-06423.2001 PMC6763177

[B20] Goodman T. Crandall J. E. Nanescu S. E. Quadro L. Shearer K. Ross A. (2012). Patterning of retinoic acid signaling and cell proliferation in the hippocampus. Hippocampus 22, 2171–2183 10.1002/hipo.22037 22689466 PMC3505796

[B21] Gould E. Tanapat P. (1999). Stress and hippocampal neurogenesis. Biol. Psychiatry 46, 1472–1479 10.1016/S0006-3223(99)00247-4 10599477

[B22] Holmes M. C. Carter R. N. Noble J. Chitnis S. Dutia A. Paterson J. M. (2010). 11beta-hydroxysteroid dehydrogenase type 1 expression is increased in the aged mouse hippocampus and parietal cortex and causes memory impairments. J. Neurosci. 30, 6916–6920 10.1523/JNEUROSCI.0731-10.2010 20484633 PMC2885438

[B23] Holmes M. C. Yau J. L. Kotelevtsev Y. Mullins J. J. Seckl J. R. (2003). 11 Beta-hydroxysteroid dehydrogenases in the brain: two enzymes two roles. Ann. N. Y. Acad. Sci. 1007, 357–366 10.1196/annals.1286.035 14993069

[B24] Hunter R. G. Bellani R. Bloss E. Costa A. McCarthy K. McEwen B. S. (2009). Regulation of kainate receptor subunit mRNA by stress and corticosteroids in the rat hippocampus. PLoS ONE 4:e4328 10.1371/journal.pone.0004328 19180187 PMC2627898

[B25] Husson M. Enderlin V. Alfos S. Boucheron C. Pallet V. Higueret P. (2004). Expression of neurogranin and neuromodulin is affected in the striatum of vitamin A-deprived rats. Brain Res. Mol. Brain Res. 123, 7–17 10.1016/j.molbrainres.2003.12.012 15046861

[B26] Husson M. Enderlin V. Alfos S. Feart C. Higueret P. Pallet V. (2003). Triiodothyronine administration reverses vitamin A deficiency-related hypo-expression of retinoic acid and triiodothyronine nuclear receptors and of neurogranin in rat brain. Br. J. Nutr. 90, 191–198 10.1079/BJN2003877 12844391

[B27] Issa A. M. Rowe W. Gauthier S. Meaney M. J. (1990). Hypothalamic-pituitary-adrenal activity in aged, cognitively impaired and cognitively unimpaired rats. J. Neurosci. 10, 3247–3254 2170594 10.1523/JNEUROSCI.10-10-03247.1990 PMC6570181

[B28] Jacobs S. Lie D. C. Decicco K. L. Shi Y. Deluca L. M. Gage F. H. (2006). Retinoic acid is required early during adult neurogenesis in the dentate gyrus. Proc. Natl. Acad. Sci. U.S.A. 103, 3902–3907 10.1073/pnas.0511294103 16505366 PMC1450163

[B29] Jiang W. Yu Q. Gong M. Chen L. Wen E. Y. Bi Y. (2012). Vitamin A deficiency impairs postnatal cognitive function via inhibition of neuronal calcium excitability in hippocampus. J. Neurochem. 121, 932–943 10.1111/j.1471-4159.2012.07697.x 22352986

[B30] Joels M. Karst H. Alfarez D. Heine V. M. Qin Y. van Riel E. (2004). Effects of chronic stress on structure and cell function in rat hippocampus and hypothalamus. Stress 7, 221–231 10.1080/10253890500070005 16019587

[B31] Joels M. Krugers H. J. (2007). LTP after stress: up or down? Neural Plast. 2007, 93202 10.1155/2007/93202 17502912 PMC1847472

[B32] Klempin F. Kempermann G. (2007). Adult hippocampal neurogenesis and aging. Eur. Arch. Psychiatry Clin. Neurosci. 257, 271–280 10.1007/s00406-007-0731-5 17401726

[B33] Krugers H. J. Lucassen P. J. Karst H. Joels M. (2010). Chronic stress effects on hippocampal structure and synaptic function: relevance for depression and normalization by anti-glucocorticoid treatment. Front. Synaptic Neurosci. 2:24 10.3389/fnsyn.2010.00024 21423510 PMC3059694

[B34] Lane M. A. Bailey S. J. (2005). Role of retinoid signalling in the adult brain. Prog. Neurobiol. 75, 275–293 10.1016/j.pneurobio.2005.03.002 15882777

[B35] Lemaire V. Lamarque S. Le Moal M. Piazza P. V. Abrous D. N. (2006). Postnatal stimulation of the pups counteracts prenatal stress-induced deficits in hippocampal neurogenesis. Biol. Psychiatry 59, 786–792 10.1016/j.biopsych.2005.11.009 16460692

[B36] Lupien S. J. de Leon M. de Santi S. Convit A. Tarshish C. Nair N. P. (1998). Cortisol levels during human aging predict hippocampal atrophy and memory deficits. Nat. Neurosci. 1, 69–73 10.1038/271 10195112

[B37] Magarinos A. M. McEwen B. S. (1995). Stress-induced atrophy of apical dendrites of hippocampal CA3c neurons: involvement of glucocorticoid secretion and excitatory amino acid receptors. Neuroscience 69, 89–98 10.1016/0306-4522(95)00259-L 8637636

[B37a] Marill J. Idres N. Capron C. C. Nguyen E. Chabot G. G. (2003). Retinoic acid metabolism and mechanism of action: a review. Curr. Drug Metab. 4, 1–10 10.2174/1389200033336900 12570742

[B38] McCaffery P. Zhang J. Crandall J. E. (2006). Retinoic acid signaling and function in the adult hippocampus. J. Neurobiol. 66, 780–791 10.1002/neu.20237 16688774

[B39] McEwen B. S. (1999). Stress and hippocampal plasticity. Annu. Rev. Neurosci. 22, 105–122 10.1146/annurev.neuro.22.1.105 10202533

[B40] Mingaud F. Mormede C. Etchamendy N. Mons N. Niedergang B. Wietrzych M. (2008). Retinoid hyposignaling contributes to aging-related decline in hippocampal function in short-term/working memory organization and long-term declarative memory encoding in mice. J. Neurosci. 28, 279–291 10.1523/JNEUROSCI.4065-07.2008 18171945 PMC6671152

[B41] Minni A. M. Dorey R. Pierard C. Dominguez G. Helbling J. C. Foury A. (2012). Critical role of plasma corticosteroid-binding-globulin during stress to promote glucocorticoid delivery to the brain: impact on memory retrieval. Endocrinology 153, 4766–4774 10.1210/en.2012-1485 22930537

[B42] Misner D. L. Jacobs S. Shimizu Y. de Urquiza A. M. Solomin L. Perlmann T. (2001). Vitamin A deprivation results in reversible loss of hippocampal long-term synaptic plasticity. Proc. Natl. Acad. Sci. U.S.A. 98, 11714–11719 10.1073/pnas.191369798 11553775 PMC58795

[B43] Mizoguchi K. Ikeda R. Shoji H. Tanaka Y. Maruyama W. Tabira T. (2009). Aging attenuates glucocorticoid negative feedback in rat brain. Neuroscience 159, 259–270 10.1016/j.neuroscience.2008.12.020 19141312

[B44] Mohler E. G. Browman K. E. Roderwald V. A. Cronin E. A. Markosyan S. Scott Bitner R. (2011). Acute inhibition of 11beta-hydroxysteroid dehydrogenase type-1 improves memory in rodent models of cognition. J. Neurosci. 31, 5406–5413 10.1523/JNEUROSCI.4046-10.2011 21471376 PMC6622712

[B45] Moisan M. P. (2010). Genotype-phenotype associations in understanding the role of corticosteroid-binding globulin in health and disease animal models. Mol. Cell. Endocrinol. 316, 35–41 10.1016/j.mce.2009.07.017 19643164

[B46] Moisan M. P. Seckl J. R. Edwards C. R. (1990). 11 beta-hydroxysteroid dehydrogenase bioactivity and messenger RNA expression in rat forebrain: localization in hypothalamus, hippocampus, and cortex. Endocrinology 127, 1450–1455 10.1210/endo-127-3-1450 2387261

[B47] Montaron M. F. Drapeau E. Dupret D. Kitchener P. Aurousseau C. Le Moal M. (2006). Lifelong corticosterone level determines age-related decline in neurogenesis and memory. Neurobiol. Aging 27, 645–654 10.1016/j.neurobiolaging.2005.02.014 15953661

[B48] Oitzl M. S. de Kloet E. R. (1992). Selective corticosteroid antagonists modulate specific aspects of spatial orientation learning. Behav. Neurosci. 106, 62–71 10.1037/0735-7044.106.1.62 1313244

[B49] Olson C. R. Mello C. V. (2010). Significance of vitamin A to brain function, behavior and learning. Mol. Nutr. Food Res. 54, 489–495 10.1002/mnfr.200900246 20077419 PMC3169332

[B50] Paez-Pereda M. Kovalovsky D. Hopfner U. Theodoropoulou M. Pagotto U. Uhl E. (2001). Retinoic acid prevents experimental Cushing syndrome. J. Clin. Invest. 108, 1123–1131 10.1172/JCI11098 11602619 PMC209498

[B51] Richard E. M. Helbling J. C. Tridon C. Desmedt A. Minni A. M. Cador M. (2010). Plasma transcortin influences endocrine and behavioral stress responses in mice. Endocrinology 151, 649–659 10.1210/en.2009-0862 20022933

[B52] Ross A. C. Russell R. M. Miller S. A. Munro I. C. Rodricks J. V. Yetley E. A. (2009). Application of a key events dose-response analysis to nutrients: a case study with vitamin A (retinol). Crit. Rev. Food Sci. Nutr. 49, 708–717 10.1080/10408390903098749 19690996 PMC2840874

[B53] Sakamuri V. P. Ananthathmakula P. Veettil G. N. Ayyalasomayajula V. (2011). Vitamin A decreases pre-receptor amplification of glucocorticoids in obesity: study on the effect of vitamin A on 11beta-hydroxysteroid dehydrogenase type 1 activity in liver and visceral fat of WNIN/Ob obese rats. Nutr. J. 10, 70 10.1186/1475-2891-10-70 21696642 PMC3142207

[B54] Sandi C. (2003). [Glucocorticoid involvement in memory consolidation]. Rev. Neurol. 37, 843–848 14606053

[B55] Sandi C. Cordero M. I. Merino J. J. Kruyt N. D. Regan C. M. Murphy K. J. (2004). Neurobiological and endocrine correlates of individual differences in spatial learning ability. Learn. Mem. 11, 244–252 10.1101/lm.73904 15169853 PMC419726

[B56] Seckl J. R. (1997). 11beta-Hydroxysteroid dehydrogenase in the brain: a novel regulator of glucocorticoid action? Front. Neuroendocrinol. 18, 49–99 10.1006/frne.1996.0143 9000459

[B57] Sooy K. Webster S. P. Noble J. Binnie M. Walker B. R. Seckl J. R. (2010). Partial deficiency or short-term inhibition of 11beta-hydroxysteroid dehydrogenase type 1 improves cognitive function in aging mice. J. Neurosci. 30, 13867–13872 10.1523/JNEUROSCI.2783-10.2010 20943927 PMC3016616

[B58] Souganidis E. Laillou A. Leyvraz M. Moench-Pfanner R. (2013). A comparison of retinyl palmitate and red palm oil beta-carotene as strategies to address Vitamin A deficiency. Nutrients 5, 3257–3271 10.3390/nu5083257 23955382 PMC3775252

[B59] Sousa N. Lukoyanov N. V. Madeira M. D. Almeida O. F. Paula-Barbosa M. M. (2000). Reorganization of the morphology of hippocampal neurites and synapses after stress-induced damage correlates with behavioral improvement. Neuroscience 97, 253–266 10.1016/S0306-4522(00)00050-6 10799757

[B60] Touyarot K. Bonhomme D. Roux P. Alfos S. Lafenetre P. Richard E. (2013). A mid-life vitamin A supplementation prevents age-related spatial memory deficits and hippocampal neurogenesis alterations through CRABP-I. PLoS ONE 8:e72101 10.1371/journal.pone.0072101 23977218 PMC3747058

[B61] Walf A. A. Frye C. A. (2007). The use of the elevated plus maze as an assay of anxiety-related behavior in rodents. Nat. Protoc. 2, 322–328 10.1038/nprot.2007.44 17406592 PMC3623971

[B62] Yau J. L. McNair K. M. Noble J. Brownstein D. Hibberd C. Morton N. (2007). Enhanced hippocampal long-term potentiation and spatial learning in aged 11beta-hydroxysteroid dehydrogenase type 1 knock-out mice. J. Neurosci. 27, 10487–10496 10.1523/JNEUROSCI.2190-07.2007 17898220 PMC6673151

[B63] Yau J. L. Noble J. Kenyon C. J. Hibberd C. Kotelevtsev Y. Mullins J. J. (2001). Lack of tissue glucocorticoid reactivation in 11beta-hydroxysteroid dehydrogenase type 1 knockout mice ameliorates age-related learning impairments. Proc. Natl. Acad. Sci. U.S.A. 98, 4716–4721 10.1073/pnas.071562698 11274359 PMC31900

[B64] Yau J. L. Noble J. Seckl J. R. (2011). 11beta-hydroxysteroid dehydrogenase type 1 deficiency prevents memory deficits with aging by switching from glucocorticoid receptor to mineralocorticoid receptor-mediated cognitive control. J. Neurosci. 31, 4188–4193 10.1523/JNEUROSCI.6145-10.2011 21411659 PMC3132450

[B65] Yau J. L. Olsson T. Morris R. G. Meaney M. J. Seckl J. R. (1995). Glucocorticoids, hippocampal corticosteroid receptor gene expression and antidepressant treatment: relationship with spatial learning in young and aged rats. Neuroscience 66, 571–581 10.1016/0306-4522(94)00612-9 7644021

[B66] Yau J. L. Seckl J. R. (2012). Local amplification of glucocorticoids in the aging brain and impaired spatial memory. Front. Aging Neurosci. 4:24 10.3389/fnagi.2012.00024 22952463 PMC3430012

[B67] Yau S. Y. Lau B. W. Zhang E. D. Lee J. C. Li A. Lee T. M. (2012). Effects of voluntary running on plasma levels of neurotrophins, hippocampal cell proliferation and learning and memory in stressed rats. Neuroscience 222, 289–301 10.1016/j.neuroscience.2012.07.019 22813995

[B68] Zhang M. Huang K. Zhang Z. Ji B. Zhu H. Zhou K. (2011). Proteome alterations of cortex and hippocampus tissues in mice subjected to vitamin A depletion. J. Nutr. Biochem. 22, 1003–1008 10.1016/j.jnutbio.2010.08.012 21190828

